# Genome-wide transcriptome analysis and identification of benzothiadiazole-induced genes and pathways potentially associated with defense response in banana

**DOI:** 10.1186/s12864-018-4830-7

**Published:** 2018-06-13

**Authors:** Zhihao Cheng, Xiang Yu, Shuxia Li, Qiong Wu

**Affiliations:** 1 Haikou Experimental Station, Chinese Academy of Tropical Agricultural Sciences, Haikou, 570102 China; 2 Department of Biology, University of Pennsylvania, Philadelphia, PA 19104 USA; 3 Institute of Tropical Bioscience and Biotechnology, Chinese Academy of Tropical Agricultural Sciences, Haikou, 571101 China

**Keywords:** Banana, Fusarium wilt, Transcriptome, Resistance inducers, Plant phytohormones, Cell wall modification

## Abstract

**Background:**

Bananas (*Musa* spp.) are the most important fruit crops worldwide due to their high nutrition value. Fusarium wilt of banana, caused by fungal pathogen *Fusarium oxysporum* f. sp. *cubense* tropical race 4 (Foc 4), is considered as the most destructive disease in the world and results in extensive damage leading to productivity loss. The widespread use of plant resistance inducers (PRIs), such as benzothiadiazole (BTH), is a novel strategy to stimulate defense responses in banana plants to protect against pathogens infection. The recent focus on the crop defense against fungal infections has led to a renewed interest on understanding the molecular mechanisms of specific PRIs-mediated resistance. This transcriptome study aimed to identify genes that are associated with BTH-induced resistance. Patterns of gene expression in the leaves and roots of BTH-sprayed banana plants were studied using RNA-Seq.

**Results:**

In this study, 18 RNA-Seq libraries from BTH-sprayed and untreated leaves and roots of the Cavendish plants, the most widely grown banana cultivar, were used for studying the transcriptional basis of BTH-related resistance. Comparative analyses have revealed that 6689 and 3624 differentially expressed genes were identified in leaves and roots, respectively, as compared to the control. Approximately 80% of these genes were differentially expressed in a tissue-specific manner. Further analysis showed that signaling perception and transduction, transcription factors, disease resistant proteins, plant hormones and cell wall organization-related genes were stimulated by BTH treatment, especially in roots. Interestingly, the ethylene and auxin biosynthesis and response genes were found to be up-regulated in leaves and roots, respectively, suggesting a choice among BTH-responsive phytohormone regulation.

**Conclusions:**

Our data suggests a role for BTH in enhancing banana plant defense responses to Foc 4 infection, and demonstrates that BTH selectively affect biological processes associated with plant defenses. The genes identified in the study could be further studied and exploited to develop Foc 4-resistant banana varieties.

**Electronic supplementary material:**

The online version of this article (10.1186/s12864-018-4830-7) contains supplementary material, which is available to authorized users.

## Background

The inability of higher plants to escape from microbial pathogen attacking has led to the development of a diverse set of constitutive and/or inducible defence mechanisms to resist and coexist with their pathogens [1]. Ongoing researches are revealing that a typical plant immune response includes a series of consecutive reactions from pathogen recognition, signal transduction, and hormone-mediated downstream defense responses [1, 2]. To recognize and cope with pathogens, plants have evolved two inducible immune systems that include pathogen-associated molecular pattern (PAMP)-triggered immunity (PTI) and effector-triggered immunity (ETI) [3]. On the external face of the host plant cell, PTI is induced when PAMPs are recognized by receptor proteins called pattern recognition receptors (PRRs). ETI is initiated by recognition of pathogen avirulence molecules called effectors by the plants disease-resistance (R) proteins [1]. Although PTI and ETI lead to similar responses, ETI is generally stronger and faster, and often gives rise to programmed cell death called the hypersensitive response (HR) and systemic acquired resistance (SAR) in the host [1].

Both PTI- and ETI-associated immune responses act through a common set of signaling components, including Ca^2+^, reactive oxygen species (ROS) and multiple protein kinases. These signaling components modulate downstream regulatory protein activities, such as transcription factors (TFs), which lead to massive transcriptional reprogramming and result in accumulation of various enzymes, pathogenesis-related (PR) proteins and pathogen infection-responsive metabolites [1]. The changes in cytosolic Ca^2+^ concentration in plant cells are believed to be one of the early intracellular reactions following pathogen perception, which are sensed by Ca^2+^-binding proteins and kinases, such as calcium dependent protein kinases (CDPKs) and mitogen-activated protein kinases (MAPKs) [2, 4]. In *Arabidopsis*, CDPKs have proved to be required for FLS2-dependent immunity [5]. Importantly, previous studies have also shown that MAPK cascade acts downstream of flagellin perception in *Arabidopsis* [6]. Moreover, transcription factors including ethylene response factors (*ERFs*) and *WRKYs* can be activated by MAPKs, and play broad and pivotal roles in regulating defenses [7, 8]. Genome-wide analyses of MAPK signaling and *WRKY* genes that are responsive to *Sclerotinia. sclerotiorum* in *Brassica. napus* have been reported [9]. ROS serves as a major signaling molecule in plant defense response [1]. A rapid production of ROS in an oxidative burst, which is largely derived from the activity of membrane-localized NADPH oxidases, was observed after infection with a pathogen in plants [10]. According to an earlier study, the expression levels of NADPH oxidases were increased in the resistant banana cultivar in response to Foc 4 infection [11], indicating the crucial role of ROS in plant defence.

In the last decades, phytohormones such as salicylic acid (SA), jasmonic acid (JA), ethylene (ET), abscisic acid (ABA) and auxin, have been extensively studied and demonstrated to play conserved and divergent roles downstream of PTI or ETI activation in defense responses [12, 13]. According to the previous studies, SA triggers defence responses against biotrophic and hemibiotrophic invading pathogens in various species, whereas JA and ET activate responses against necrotrophic pathogens [14]. It has been found that SA accumulation and signaling is enhanced during plant-pathogen interactions in many species [14–16]. In tomato mutants with impaired in SA accumulation and perception exhibited increased susceptibility against to *Fusarium* pathogen infection, indicating the importanceof SA in plant defence [17]. Exogenous application of SA or synthetic SA analogs, such as benzothiadiazole (BTH), potentiated responses to various pathogens in a wide range of tested plants including *Arabidopsis*, tomato, wheat and cucumber [18–21]. The non-expressor of pathogenesis related gene 1 (NPR1) is an essential component of SA-induced signaling pathway and SAR. It was observed that *NPR1A* and *NPR1B* were strongly induced by Foc infection in banana [22]. Consistent with the defense promoting role of SA, ET and JA have also been demonstrated to fine-tune immunity in plant-pathogen interactions [14]. Mutations in Ethylene receptor 1 (ETR1) and Ethylene-insensitive 2 (EIN2), two essential components of ET perception and transduction, showed a reduction of disease symptoms compared to the wild-type upon *Fusarium oxysporum* f.sp. *conglutinans* (Focn) or *Fusarium oxysporum* f.sp. *raphani* (Forp) inoculation in *Arabidopsis* [23, 24]. Accordingly, loss-of-function mutations in Coronatine-Insensitive 1 (COI1) that are required for JA-mediated defense also resulted in strongly increased resistance to Focn [25]. In contrast to the hormones aforementioned, the role of auxin in modulating the defense pathways has not been systematically analyzed. Previously, auxin was believed to be involved in mediating plant growth and organogenesis [26, 27]. Recently, the altered accumulations of auxin in plant-pathogen interaction suggests that this hormone may play an important role in plant defence [28, 29].

Fusarium wilt, which is mainly caused by the fungal pathogen *Fusarium oxysporum* f. sp. *cubense* tropical race 4 (Foc 4), is a soil-borne lethal disease of Cavendish bananas in the world [2, 30, 31]. The Foc 4 infects host banana plants through attaching to the root surface, penetrating and subsequent colonizing the xylem vessels, and causes a reddish-brown discoloration of the rhizome and pseudostem [32]. Typical disease symptoms of Fusarium wilt mainly include progressive wilting; yellow leaves followed by stunting and eventually plant death [30]. Susceptible banana cultivars could not be produced in previously infested plantations for a long period of time, up to 30 years, due to the pathogen’s long persistence in the absence of the host [30]. Since the 1990s, Foc 4 emerged and spread rapidly in the tropics of Africa and Asia, and was considered as a major threat to Cavendish cultivars production worldwide [2]. Previous studies indicated that a diverse range of methods can be used to combat or manage this disease [30]. Among them, identification of new cultivars by resistance breeding remains the most promising option to overcome the Fusarium wilt of banana plants. However, identification of resistant cultivars is time consuming and sometimes is hindered by low seeds production of triploid banana. Importantly, application of nontoxic ‘resistance inducer’ is also a preferred method to control plant diseases through induction of plants’ defense mechanisms [33]. A great number of exogenous molecules have been effectively used against pathogen in many species, such as β -aminobutyric acid (BABA), hexanoic acid and BTH [33]. BTH has been reported to be effective in the management and prevention of pathogen infection through activating SAR genes that encode PR proteins, ROS scavenger enzymes and the chitinase in several species [18, 34, 35], but it has not been shown to protect banana against Foc 4 infection until now. In this study, we found that spraying of banana plants (Cavendish cultivars) with BTH reduce disease incidence and severity caused by Foc 4, revealing the ability of BTH to induce the banana plant immune system. However, how exactly BTH levels influence banana defense to Foc 4 remains to be addressed. RNA sequencing (RNA-Seq) analysis is a powerful approach to study transcriptomes, and has led the way to identify BTH-responsive signaling pathways tightly linked to Fusarium wilt that are highly valued in breeding. Importantly, previous studies mainly focused on studying the molecular mechanism of susceptible banana infected with Foc 4 [11, 36–39], a global analysis of the transcriptome responses associated with resistance inducer has not yet been performed. Therefore, to determine the effects of BTH on plant metabolism, we analyzed the transcriptome of Cavendish banana leaves and roots treated with BTH. Our study helped us to understand BTH-mediated host defenses against Foc 4 in banana and to formulate strategies to protect plants by using resistance inducers.

## Methods

### Plant material and Foc 4 inoculations

Cavendish banana (*Musa* spp. AAA group) was used in the study. Two-month-old seedlings were grown in plastic pots filled with sterilized soil at 26 ± 2 °C with a 16 h/8 h light/dark period and with 80–90% relative humidity in greenhouse of Chinese Academy of Tropical Agricultural Sciences, Haikou of China (N20°02′and E110°11′). The BTH treatment was performed by spraying the leaves of the banana plant with the 50 mg/L, 100 mg/L BTH. Plants sprayed with distilled water were used as the negative controls. For inoculation, the Foc 4 was used to inoculate roots as previously described [32], and at least 20 individual plants for each treatment of three replicated assays were phenotypically screened at 30 days post inoculation. Fusarium wilt disease was monitored based on the observation of typical wilt symptoms, and the disease incidence was calculated as the percentage of infected plants among the total number of plants. The differences of disease incidence between groups were determined by one-way analysis of variance (ANOVA) as previously described [40]. For RNA-sequencing, six individual banana seedlings planted in pots were sprayed with 100 mg/L BTH, and the youngest leaves and roots were collected at 1st day post treatment (dpt) and 3 dpt, respectively, and then were frozen in liquid nitrogen immediately. Based on the previous studies, the transcripts level of SAR markers and the number of BTH-responsive genes were increased gradually and reached the peak at 24 h after BTH treatment [18, 41, 42]. Meantime, we speculated that the effect of BTH on banana transciptome may also be different over the time, and we proposed two types of response, including short-term transient response (1 day) and long-term response (3 days). Thus, we choose these two sampling time points. For each sampling time and treatment, three replicated samples were collected from both the treated and control plants.

### RNA-Seq and data analysis

For RNA-Seq, the total RNA isolation, cDNA libraries preparation and RNA deep sequencing were performed by the Annoroad Gene Technology Corporation (Beijing, PR China). A total of 105 gigabase raw sequence reads of 18 libraries was obtained initially on a HiSeq 2500 instrument that generated paired-end reads with 150 nucleotides in length. We discarded reads with adapters, those with more than 5% unknown nucleotides, and those of low quality (≥ 15% of the bases with a quality score (Q) ≤ 19). The average proportion of clean reads in each sample was 88.5–90.2%. The filtered clean reads from each sample were aligned to the reference banana genome with mapping rate 73–78%, and were assembled into non-redundant unigenes using Cufflinks v2 method [43]. The FPKM (fragments per kilo base of transcript per million mapped reads) was employed to estimate the abundance of unigenes [44]. The differential expression analysis between treatments and controls was performed using cuffdiff program, and the significant DEGs for further data analysis were filtered with false discovery rate (FDR) corrected *P* value ≤0.05 and fold change ≥2. The RNA-Seq data was submitted to BioProject in NCBI under the accession number PRJNA417328.

### Functional annotation of DEGs and pathways

To obtain functional annotation and identify the putative biological pathways of DEGs, we annotated the DEGs using the NR protein database (NCBI), GO and KEGG databases with similarity set at > 30% and an E-value ≤1e^− 5^. As described previously [45], The GO seq R package was employed to attain GO-based classification and pathway mapping [46]. KOBAS software was used to calculate the statistical enrichment of DEGs in KEGG pathway [47]. The FDR was used to determine the threshold of the *P*-value in GO and KEGG analyses. We used an FDR of < 0.05 as thresholds to define significantly enriched GO terms and KEGG pathways.

### Real-time quantitative PCR verification

Total RNA was isolated from the leaf and roots of banana plants sprayed with 100 mg/L BTH, as described above. First-strand cDNA synthesis was performed using PrimeScript™ RT reagent Kit (Takara) according to the manufacturer’s instructions, using 2 μg total RNA and oligo(dT) primers. qRT-PCR was performed using SYBR® qPCR Mix (Toyobo, Tokyo, Japan) according to the manufacturer’s protocol. The cycling conditions were as follows: 5 min denaturation at 95 °C, followed by 40 cycles at 95 °C for 15 s, and 60 °C for 40 s. Melting curve analysis was performed over the range of 65 to 98 °C. The primers used in this study were listed in Additional file 1: Table S7. All qRT-PCR reaction was repeated three times and the relative mRNA expression level was calculated as 2^-ΔΔCt^. The banana *MaACTIN* gene was used as a constitutive reference. The qPCR data were presented as means ± SD. Asterisks indicate significant differences compared with the control by Student’s t-test.

## Results

### Chemically induced resistance in banana against Foc 4

**Fig. 1:**
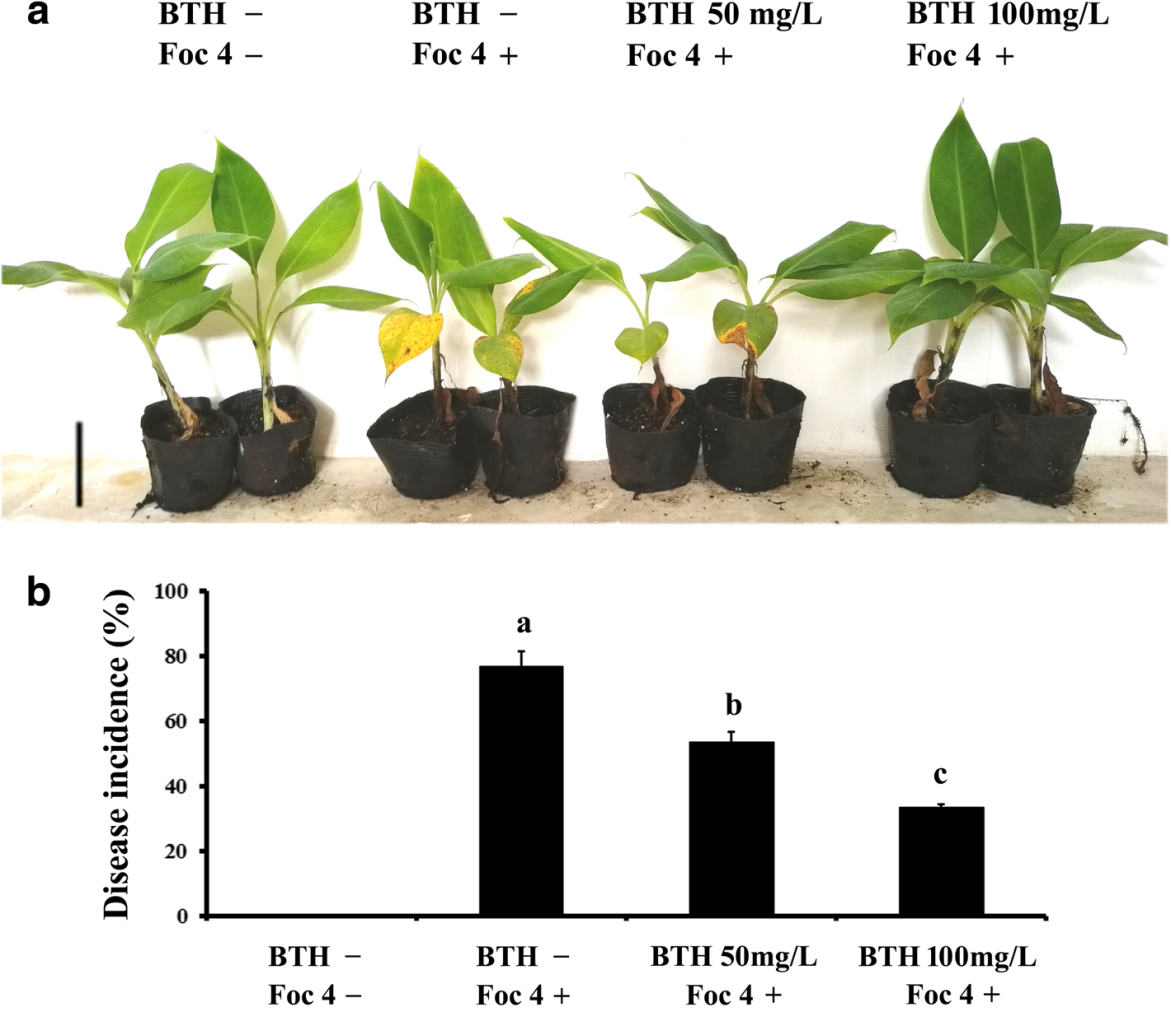
Growth of banana plants following treatment with BTH and Foc 4 inoculation. **a** Two-month-old banana plants (with four true fully expanded leaves) were pre-treated with H_2_O, 50 mg/L BTH or 100 mg/L BTH. The roots were infected with Foc 4 or untreated one day later. Plants were photographed 4 weeks post inoculation; **b**) Fusarium wilt disease incidence rate (calculated by the formula: number of infected plants/ total number of inoculated plants) of Foc 4 inoculated or untreated plants. Data are presented as mean ± SD of three independent assays, and the different letters indicate significant differences (*P* < 0.05) according to ANOVA test. The total number of plants observed was > 20 in each group from three biological replicates

BTH has already been documented to be an identical efficient activator of disease resistance in several plant species [18, 34, 35]. However, little is known about whether BTH is effective against Fusarium wilt caused by Foc 4 in Cavendish bananas. To evaluate its activity on banana, leaves of two-month-old plants (with four true leaves) were sprayed with water or BTH, 1 day prior to inoculation of the roots with Foc 4. Based on previously reported BTH concentrations for foliar treatments in different plants, 50 mg/L and 100 mg/L BTH treatments were employed. These concentrations had no visible effect on banana plant development. Thirty days after inoculation, the disease severity was analyzed statistically. As shown in Fig. 1, more than 76.6% of control plants inoculated with Foc 4 exhibited very severe symptoms, while almost 70.1% of Foc 4-infected plants pre-treated with BTH were symptomless. Symptoms in control plants consisted of leaf yellowing, and as the disease progresses, a pronounced stunting of the whole plants was observed. The results indicated that BTH is able to enhance banana resistance to Foc 4 without affecting the growth of banana seedlings.

### Overview of banana transcriptome after BTH treatment

To achieve a broad survey of new candidates important in BTH-mediated plant defense response, RNA-Seq data were generated from banana leaves and roots sampled 1 and 3 days from plants leaves sprayed with BTH, as compared to the control. At least three replicates for each treatment were harvested. Using an Illumina paired-end sequencing platform, over one billion reads from BTH-treated and control libraries were produced. After removing low quality reads, 43 million to 47 million reads per sample were mapped against the reference *Musa* spp. genome and assembled into 38,995 transcripts (Table 1). By comparing these transcripts with the annotated genes in the banana genome, an average of 89.7% of total transcripts matched the predicted banana protein-coding sequences and 34,999 non-redundant unigenes were obtained. Notably, after filtering out low-abundance (FPKM < 1) and noncoding genes, a total of 3996 protein-coding transcripts that showed no overlap with any annotated genes, were characterized as reference-dependent novel potential protein-coding genes (Table 1, Additional file 2: Table S1).

**Table 1:** Summary of the read numbers aligned onto the banana reference genome

Samples	Total Reads	Mapped Reads	Mapping Rate	Multi Map Reads	Annotated transcripts	Novel transcripts
LF 0d	61,783,609	47,826,265	0.77	339,914	32,340	2802
LF 1d	62,428,631	46,940,149	0.75	533,631	32,009	2754
LF 3d	60,616,740	47,198,078	0.78	424,275	32,081	2163
RT 0d	60,117,134	43,751,447	0.73	908,342	33,176	3370
RT 1d	60,981,186	46,515,067	0.76	509,560	33,750	3219
RT 3d	61,128,012	44,891,051	0.73	634,339	33,437	3161
In total	1,101,165,940	831,366,175	0.75	10,050,186	34,999	3996

### Differentially expressed genes responsive to BTH spraying in banana plants

**Fig. 2:**
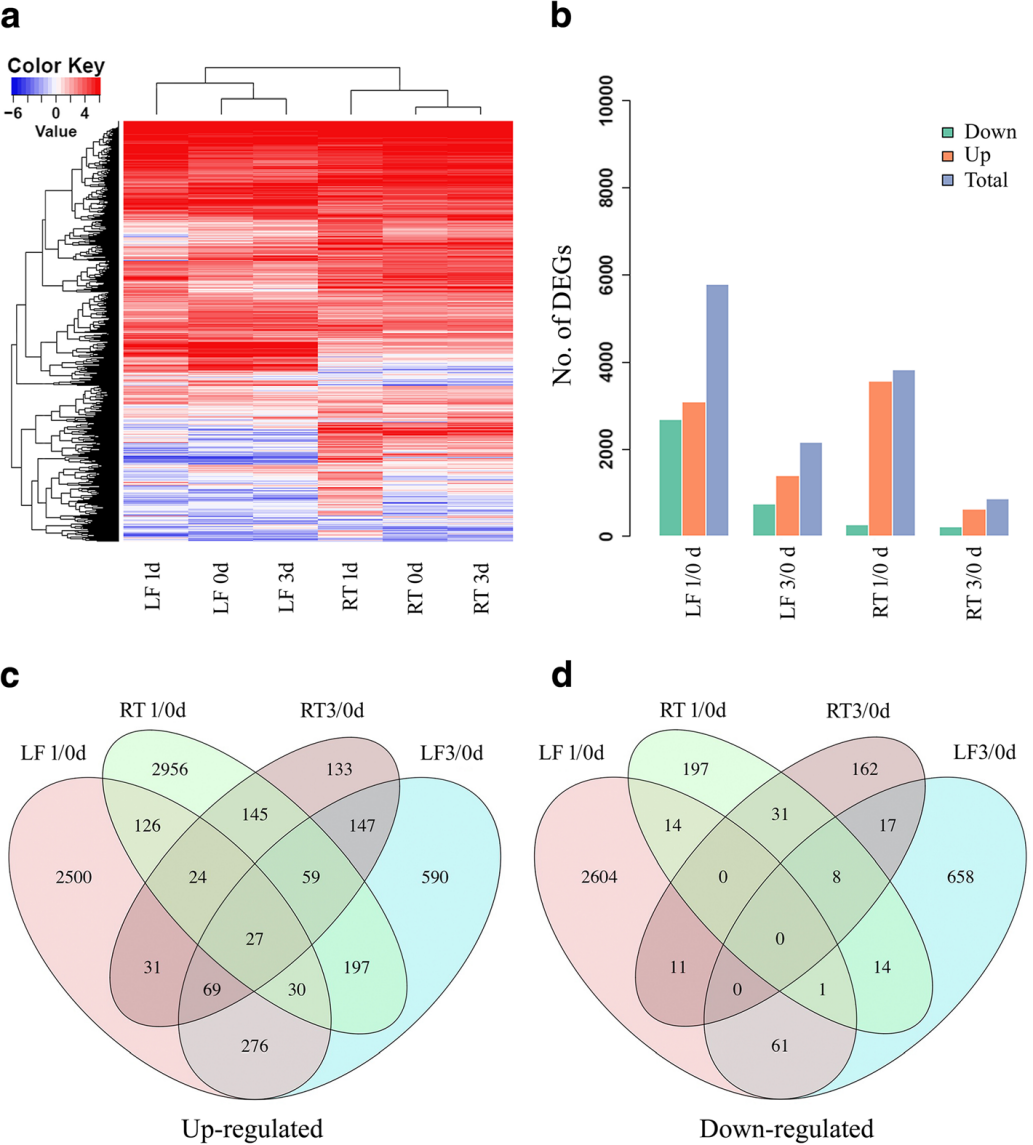
Variation in gene expression of BTH-sprayed banana plants. **a** The abundance of all of the expressed genes in leaves and roots; **b**) Comparison of the number of up- and down-regulated DEGs between treatments and control; **c**, **d**) Venn diagram represents the overlapping up- and down-regulated DEGs number between leaves and roots, respectively. LF, leaf; RT, root

To evaluate the expression value of 34,999 annotated banana protein-coding genes for each sample, the number of clean reads that mapped to each gene was calculated, and then normalized into FPKM (fragments per kb exon model per million mapped fragments). By analyzing the expression patterns of these genes in leaves and roots for each time point, we observed a large number of genes that exhibited highly dynamic changes after 1 day BTH treatment in both leaves and roots (Fig. 2a). Furthermore, the expression changes of each gene in BTH treated leaves and roots in comparison to untreated control were investigated. We used a stringent value of FDR < 0.05 as the threshold to judge the significant differences in the gene expressions. Across all of the times tested, a total of 8621 differentially expressed genes (DEGs) were identified after BTH treatment in banana (Additional file 3: Table S2). More specifically, 6689 and 3624 DEGs were significantly modulated by BTH in leaves and roots, respectively. The numbers of DEGs for each time point were shown in Fig. 2b and Additional file 3: Table S2. Among the up-regulated genes, at all time points, 46.1 and 44.4% of the genes were specifically modulated by BTH in leaves and roots, respectively (Fig. 2c). Among the down-regulated genes, 87.9 and 10.3% of the genes were uniquely altered by BTH in leaves and roots, respectively (Fig. 2d). These results indicated that most of the DEGs were responsive to BTH spraying in a tissue-specific manner.

### Functional sanalysis of BTH-responsive DEGs

To functionally classify those BTH-responsive DEGs, gene ontology (GO) enrichment analysis of all the DEGs was utilized, and a total of 298 GO terms were significantly enriched (Additional file 4: Table S3). In Table 2, we listed the top 5 specific and common GO terms enriched in leaves and roots, respectively. After 1 day BTH treatments, DEGs involved in organonitrogen compound metabolic, pigment metabolic and chlorophyll metabolic processes were significantly enriched in leaves. While the cell wall organization or biogenesis and external encapsulating structure organization processes were identified in DEGs of roots. Additional analysis revealed that DEGs associated with single-organism biosynthetic or metabolic and small molecule biosynthetic processes were commonly overexpressed in both leaves and roots. At the third day after BTH spraying, there was differential modulation on the processes including DNA-templated transcription, RNA biosynthetic and heterocycle biosynthetic in leaves. In contrast, DEGs involved in cellular response to oxygen-containing compound, response to oxygen-containing compound and jasmonic acid mediated signaling pathway were obviously affected in roots. Notably, a large number of DEGs related to biotic and abiotic stimulus responding were significantly enriched in both leaves and roots.

**Table 2:** GO terms of biological process classification of DEGs in leaves and roots

LF-specific 1/0d	GO ID	Up	Down	RT-specific 1/0d	GO ID	Up	Down
Organonitrogen compound metabolic	GO:1901564	148	261	Cell wall organization or biogenesis	GO:0071554	229	3
Pigment metabolic process	GO:0042440	27	65	Cell division	GO:0051301	184	5
Chlorophyll metabolic process	GO:0015994	15	35	External encapsulating structure organization	GO:0045229	198	3
Small molecule metabolic process	GO:0044281	214	361	Carbohydrate metabolic process	GO:0005975	365	8
Photosynthesis	GO:0015979	10	84	Cell cycle	GO:0007049	208	5
Common 1/0d							
Single-organism metabolic process	GO:0044710	456	664			745	39
Small molecule biosynthetic process	GO:0044283	82	149			158	8
Carbohydrate biosynthetic process	GO:0016051	49	80			102	1
Organic hydroxy compound biosynthetic	GO:1901617	23	45			54	3
Lipid metabolic process	GO:0006629	128	177			202	12
LF-specific 3/0d	GO ID	Up	Down	RT-specific 3/0d	GO ID	Up	Down
DNA-templated transcription	GO:0006351	221	128	Jasmonic acid mediated signaling pathway	GO:0009867	19	4
RNA biosynthetic process	GO:0032774	221	128	Cellular response to oxygen-containing compound	GO:1901701	47	14
Heterocycle biosynthetic process	GO:0018130	242	139	Defense response to bacterium	GO:0042742	29	5
Aromatic compound biosynthetic process	GO:0019438	261	142	Cellular response to jasmonic acid stimulus	GO:0071395	19	4
Organic cyclic compound biosynthetic	GO:1901362	267	145	Cellular response to acid chemical	GO:0071229	33	12
Common 3/0d							
Response to organonitrogen compound	GO:0010243	54	9			34	3
Response to endogenous stimulus	GO:0009719	194	69			121	29
Response to oxygen-containing compound	GO:1901700	162	50			106	26
Defense response	GO:0006952	141	31			69	22
Response to organic substance	GO:0010033	200	78			126	30

To further understand the functions of DEGs, we mapped all of DEGs in the kyoto encyclopedia of genes and genomes (KEGG) database to discover those genes involved in metabolic or signal transduction pathways. We identified 15 and 25 pathways that were significantly enriched in leaves and roots, respectively, which were in agreement with the GO terms results (Additional file 5: Table S4). Table 3 presents the overall response pathways of banana leaves and roots to BTH treatments at two time points, respectively. We observed that most of the pathways (12/15 in leaves and 20/25 in roots) were involved in various metabolic pathways across all of the times tested, including amino acid, nucleotide-sugar, steroid, sucrose, linoleic acid and biosynthesis of other secondary metabolites. More importantly, one common feature was also observed in both leaves and roots: plant-pathogen interaction pathway was notably enriched at the 3rd day post treatment (dpt). A few differences between the two tested tissues were also observed: cell cycle and plant hormone signal transduction pathways were specifically enriched in roots. Taken together, these results pointed, for the first time, a role of BTH in promoting pathogen resistance, especially in roots, by modulating differential gene transcription globally.

**Table 3:** The significantly enriched pathways of DEGs in banana leaves and roots

LF-specific 1/0d	Map ID	Up	Down	RT-specific 1/0d	Map ID	Up	Down
Selenocompound metabolism	map00450	2	11	Amino and nucleotide sugar metabolism	map00520	56	0
Carbon fixation in photosynthetic organisms	map00710	5	25	Phenylpropanoid biosynthesis	map00940	42	2
Cysteine and methionine metabolism	map00270	10	17	Steroid biosynthesis	map00100	18	0
Purine metabolism	map00230	8	46	Phenylalanine metabolism	map00360	34	1
Porphyrin and chlorophyll metabolism	map00860	3	14	Cell cycle	map04110	34	0
Sulfur metabolism	map00920	4	9	Drug metabolism - cytochrome P450	map00982	12	1
Common 1/0d							
Biosynthesis of secondary metabolites	map01110	87	146			189	7
Glycine, serine and threonine metabolism	map00260	5	24			18	0
Metabolic pathways	map01100	150	279			295	12
Microbial metabolism in diverse environments	map01120	26	76			60	1
Starch and sucrose metabolism	map00500	31	27			48	1
LF-specific 3/0d	Map ID	Up	Down	RT-specific 3/0d	Map ID	Up	Down
Starch and sucrose metabolism	map00500	13	11	Plant hormone signal transduction	map04075	20	3
Amino and nucleotide sugar metabolism	map00520	15	6	Phenylpropanoid biosynthesis	map00940	7	6
				Biosynthesis of secondary metabolites	map01110	26	15
				Phenylalanine metabolism	map00360	6	3
Common 1/0d							
Plant-pathogen interaction	map04626	30	3		map04626	18	1

### BTH-responsive DEGs related to signal perception and transduction

**Fig. 3:**
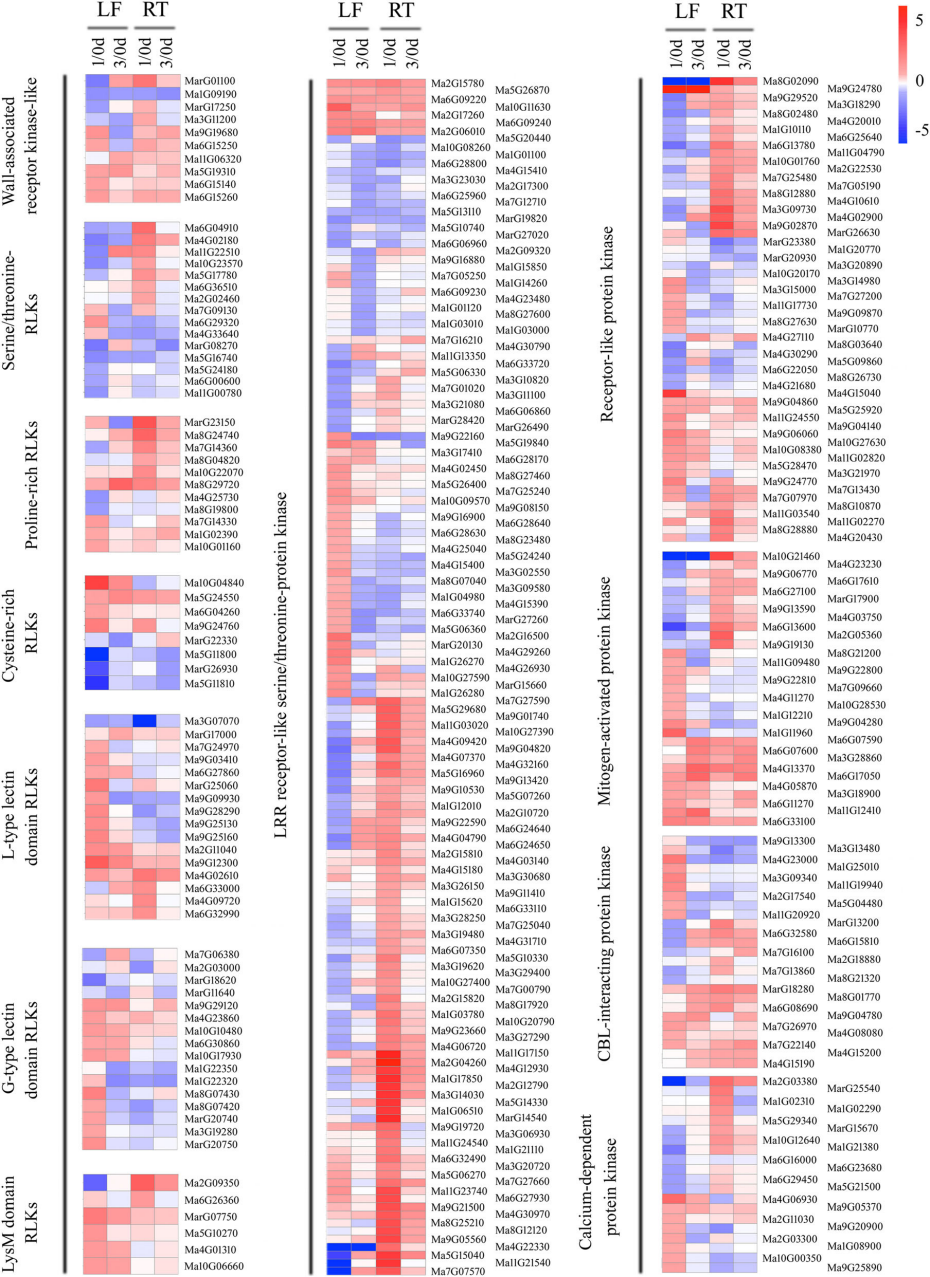
Expression pattern of banana protein kinases in response to BTH treatment. Heatmaps were generated from the fold change values of wall-associated receptor kinase-like, serine/threonine RLKs, proline-rich RLKs, cysteine-rich RLKs, L/G-type lectin domain RLKs, lysM domain RLKs, LRR-RLKs, receptor-like protein kinase, mitogen-activated protein kinase, CBL-interacting protein kinase and calcium-dependent protein kinase family numbers in leaves and roots from BTH-sprayed plants as compared to the control condition. LF, leaf; RT, root

Ongoing research is revealing that the protein kinases act as master regulators in plant adaptions to biotic stresses [48, 49]. In total, 545 protein kinases were identified to be responsive to BTH treatment, including 77 overlapping DEGs in all tissues, 287 leaves-specific and 181 roots-specific DEGs. The majority of these proteins were up-regulated in both the leaves and roots (Additional file 6: Table S5). Based on the roles of these kinases in signal perception and transduction pathways, we classified these genes into several groups, including receptor-like kinases (RLKs), serine/threonine-protein kinases, MAPKs and Ca^2+^ sensors. The RLK group has been shown to be involved in defense/resistance against pathogens in model plants [49]. A total of 30 common signaling-related receptor kinases and RLKs that responded to BTH in both leaves and roots at the early response phase were identified. These genes mainly included 3 wall-associated receptor kinase-like proteins, 2 serine/threonine-RLKs, 4 proline-rich RLKs, 3 cysteine-rich RLKs, 5 lectin domain RLKs, 2 lysM domain RLKs and 11 leucine-rich repeat (LRR)-RLKs (Fig. 3, Additional file 6: Table S5). Moreover, we also identified 7 MAPKs, 6 CBL-interacting protein kinases and 1 calcium-dependent protein kinases (CDPKs), which were accumulated to a higher level by BTH in all tissues. These results indicated that early signal perception and transduction may be enhanced in BTH-treated banana plants.

### Identification of BTH-regulated transcription factors

**Fig. 4:**
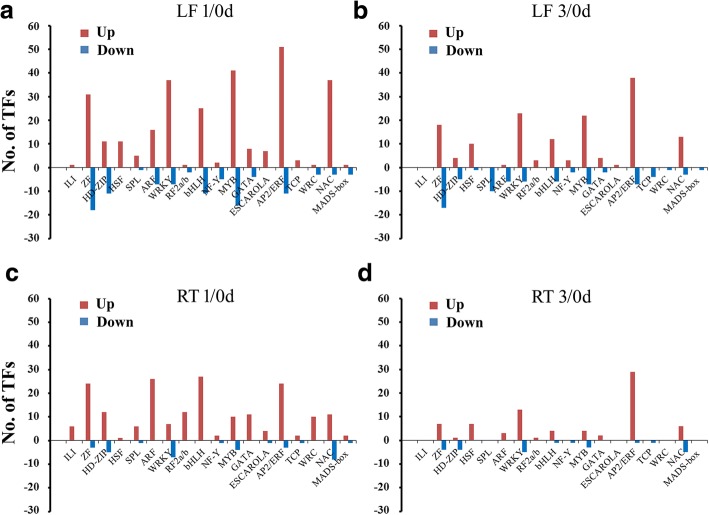
Differentially expressed DEGs representing diverse families of transcription factors in leaves and roots. **a-d**) Comparison of the number of up- and down-regulated TFs in leaves (**a**, **b**) and roots (**c**, **d**) at the two time points, respectively. LF, leaf; RT, root

Transcription factors (TFs) play major roles in plant development and defense response through the temporary and spatial inducing or repressing the transcription of their target genes [50]. In this study, a total of 946 TFs were identified to be responsive to BTH in leaves and roots, which were mainly assigned to 54 different families (Additional file 6: Table S5). Among these TFs, around 50.7% (480) of the up-regulated and 23.4% (221) of the down-regulated TFs were identified in leaves and the top four significantly changed TF families were APETALA 2/Ethylene-Responsive Factor (AP2/ERF), followed by MYB, WRKY and zinc finger (ZF) family (Fig. 4a, b). In addition, a number of development-related TFs belonging to HD-ZIP, TCP and squamosa promoter binding-like protein (SPL) families were down-regulated in leaves, indicating the developmental processes of shoots may have been inhibited temporarily by BTH. However, TFs that differentially expressed in roots exhibited a diverse trend. In addition, we found approximately 36.2% of TFs were BTH-induced, while only 7.0% (66) of them were suppressed by BTH. Particularly, the DEGs belonging to the auxin response factor (ARF) and basic helix-loop-helix (bHLH) gene families were specifically accumulated in roots, which indicated that these TFs respond to BTH more actively in roots rather than in shoots (Fig. 4c, d). Meanwhile, the number of up-regulated TFs was much larger than the number of down-regulated ones in roots, suggesting that transcriptional activation may be dominant over repression. Previous studies have shown that large families of TFs, such as AP2/ERF, WRKY, ZF and bHLH were response to developmental and environmental stimuli, and function downstream of the hormones, biotic, and abiotic stress signaling pathways [51–53]. For example, in grape (*Vitis vinifera*), 57% (16 genes) of WRKY genes showed altered expression after biotic stress induced by a common fungal pathogen (*Coniothyrium diplodiella*) infection [54]. In pepper, CaWRKY27 protein positively regulates the stress resistance response to *Ralstonia solanacearum* infection through modulation of SA-, JA- and ET-mediated signaling pathways in tobacco [55]. The ERF proteins involved in defence responses against pathogen infection have also been extensively documented, and overexpression of *ERF* genes in transgenic *Arabidopsis* or tobacco plants induces expression of several PR and hormone-responsive genes, resulting in enhanced resistance to pathogen [56, 57]. Therefore, in this study, the different expression patterns of these TFs in leaves and roots suggested a distinct requirement for diverse developmental responses in the two tissues of BTH-sprayed plants.

### Tissue-dependent cell cycle genes in the BTH response

**Fig. 5:**
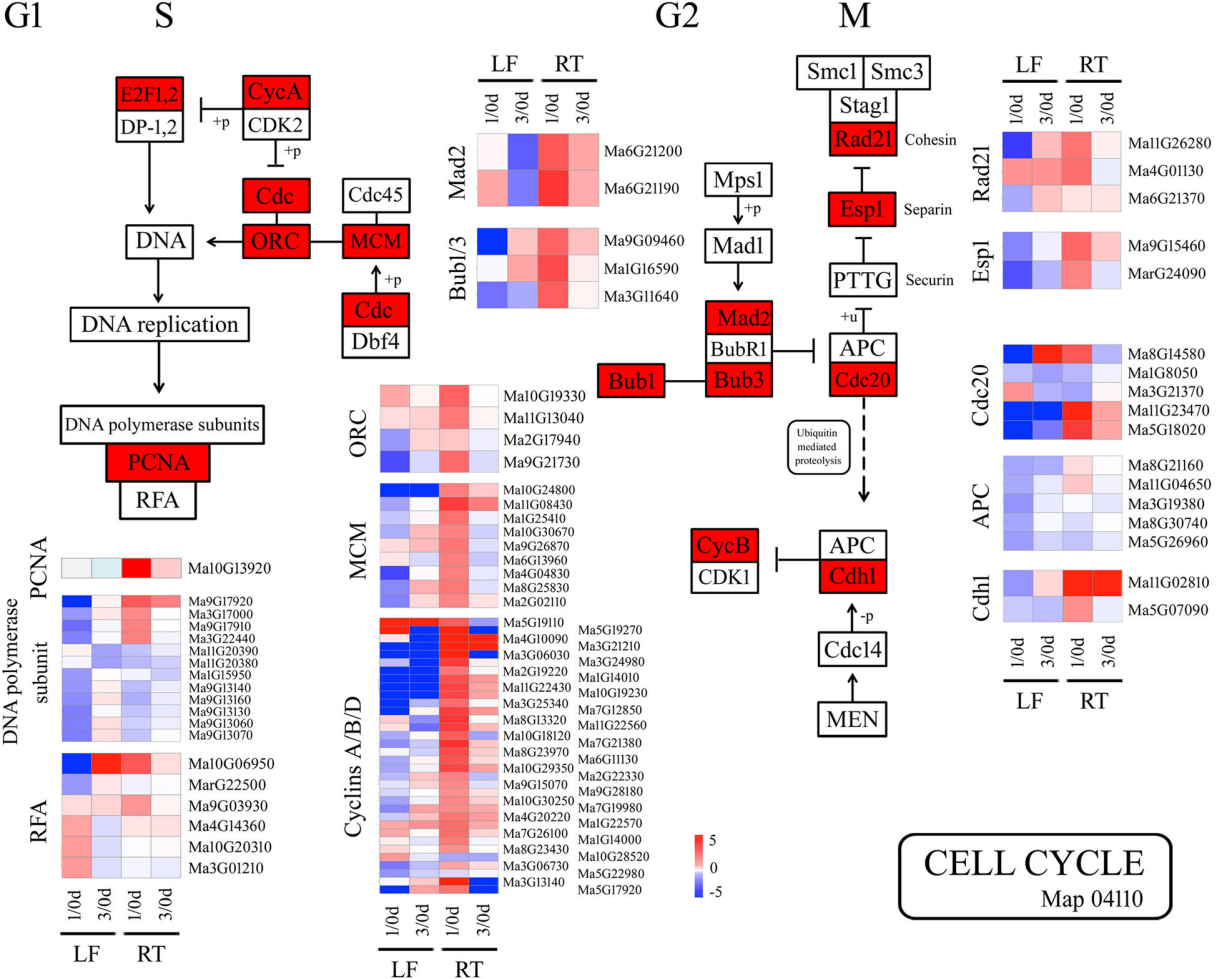
Genes with significantly changed abundances as classified into putative cell cycle control group. Heatmaps were generated from the fold change values of DEGs related to cell cycle control in leaves and roots at 1 and 3 dpt. LF, leaf; RT, root

KEGG analysis revealed the induction of 34 DEGs encoding for cell cycle-related proteins and enzymes in roots at the early time point, while no significant enrichment was observed in leaves confirming the tissue-dependent involvement of cell cycle pathway in the BTH response. In Fig. 5 and Additional file 7: Table S6, the selected DEGs associated with cell cycle are reported, which mainly included 2 mitotic spindle checkpoint proteins (MADs), 3 mitotic spindle checkpoint proteins (Bubs), 4 Origin recognition complex subunits (Orc), 9 DNA replication licensing factors (MCMs), 3 cohesions, 2 separins, 5 cell division cycle 20 (Cdc20), 1 proliferating cell nuclear antigen (PCNA) and 34 cyclins. Precise cell-cycle control is critical for plant responses to pathogen invasion [58]. Previous study has demonstrated that the tomato B-type cyclin gene, *SlCycB2*, play a critical role in reproductive organ development, trichome initiation and *Prodenia litura* defense [59]. The increases in the abundances of DNA replication- and cell cycle-related DEGs might result in accelerating the root cell division rate under treatment, which may be an important defense reaction in BTH-treated banana against Foc 4.

### BTH induces synthesis and response of plant phytohormones

**Fig. 6:**
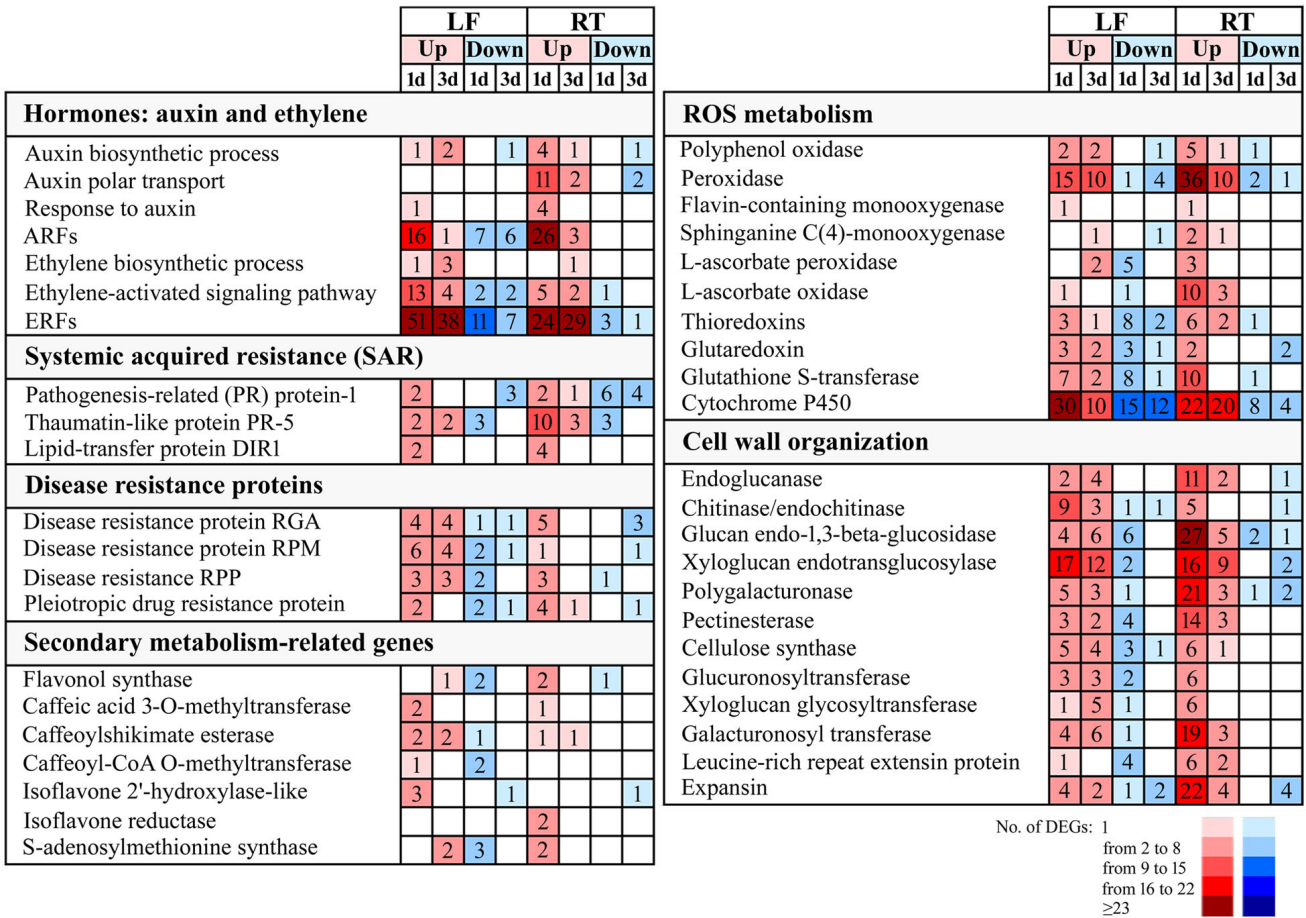
Selection of up-regulated and down-regulated DEGs in banana leaves and roots following treatment with BTH at 1 and 3 dpt. The numbers of DEGs differently expressed are reported for each gene category. LF, leaf; RT, root

To determine whether BTH influenced the hormone signaling pathways, we analyzed the expression changes of auxin, ABA, JA, SA and ET biosynthesis- and response-related genes in the leaves and roots of banana. In roots, BTH treatment significantly altered the expression of 21 genes involved in auxin signaling pathways, including auxin biosynthesis, polar transport and auxin-responsive genes (Fig. 6, Additional file 7: Table S6). For example, YUCCA protein, which encodes an indole-3-pyruvate monooxygenase, was identified as the crucial component of the auxin biosynthetic process. We identified three *YUCCAs*, such as Ma8G00130, Ma3G08830 and Ma11G12150, were up-regulated by BTH in roots. Similarly, Two PIN proteins (Ma7G17140 and Ma8G29780), which mainly function as auxin efflux carriers, were also triggered by BTH in roots. However, the majority of these auxin-related DEGs (80.9%) were found to be down-regulated or unchanged during the two stages in leaves. Oppositely, genes involved in ET biosynthetic and signaling transduction processes were predominantly increased in leaves, and only a few of these genes were affected at both time points in roots (Fig. 6, Additional file 7: Table S6). For instance, Four ET biosynthesis genes, ACC synthase (*ACS*, MarG20420, MarG26960, Ma3G02870 and Ma4G29150) were increased significantly after BTH treatment. Moreover, one ethylene receptor (*ETR*, Ma8G14350) and two EIN3-binding F-box proteins (*EBF,* Ma9G28510 and Ma4G30680), which play a significant role in perceiving and transducing the ET signals, were also induced specifically in leaves but not in roots in response to BTH-spraying (Additional file 7: Table S6). Contrary to activation of auxin and ET signaling pathways, JA and SA biosynthetic- and signaling-related genes showed no significant differences between BTH-treated and untreated plants in our results.

### Identification of SAR-related genes modulated by BTH

In recent years, SAR, an inducible defense mechanism in plants against pathogens, has been demonstrated to be associated with the activation of SA signaling [60]. Several crucial components putatively involved in SAR signaling have been identified, including lipid-transfer protein DIR1 (DEFECTIVE IN INDUCED RESISTANCE 1) and PR proteins (Fig. 6). In the present study, although the SA-related signaling pathway was unaffected, 29 PRs and 4 DIR1 genes were modulated by the BTH, respectively (Fig. 6, Additional file 7: Table S6). In particular, up-regulated genes were prevalent at 1 dpt in both the tested tissues from the banana plants, indicating that the SAR signaling may be strongly induced at this time point.

### Plant disease resistance protein induced by BTH

Plant disease resistance (R) proteins have been widely documented to play specific roles in plant-pathogen interaction through recognizing the effector molecules [2]. In the study, BTH induced up-regulation of several disease resistance proteins, which were related to pleiotropic drug resistance protein, disease resistance RPP, RPM and RGA (Fig. 6, Additional file 7: Table S6). Meanwhile, a wide variability of gene expression was also observed in BTH-sprayed leaves, and only a few of these genes were affected at the two time points in roots. Interestingly, the numbers of up-regulated and down-regulated genes were similar at 1 dpt in leaves, while for roots, a greater number of BTH-induced than repressed genes at 1 dpt (Fig. 6, Additional file 7: Table S6). These genes with significantly changed abundance might be related to disease defense and could lead to a better understanding of the mechanisms involved in BTH-induced Foc 4 responses.

### Secondary metabolism-related genes modulated by BTH

Some enzymes associated with secondary metabolism play key roles in the synthesis of antifungal compounds and cell wall components, which are involved in the phenylpropanoid pathway and other pathways. In our study, more than 27 DEGs involved in the phenylpropanoid and other secondary metabolism pathways were modulated by BTH, such as *flavonol synthase caffeic acid* (*FLS*), *caffeic acid 3-O-methyltransferase* (*COMT*), *caffeoyl-CoA O-methyltransferase* (*CCOMT*), *caffeoylshikimate esterase* (*CSE*), *isoflavone reductase* (*IFR*) and *leucoanthocyanidin dioxygenase* (*LDOX*) (Fig. 6, Additional file 7: Table S6). *COMT*, *CCOMT* and *CSE* are associated with lignin biosynthesis, which is beneficial for the lignification of the cell wall in response to pathogen infection. Several these enzymes, such as Ma11G07580, Ma10G26780 and MarG09770 were intensely up-regulated by BTH. The IFR was mainly involved in catalyzing the synthesis of isoflavonoid phytoalexin medicarpin, and was found to be rapidly induced in response to fungal pathogen infection in alfalfa [61]. We identified two *IFRs* (Ma2G14320 and Ma4G24530) were induced by BTH in roots (Fig. 6, Additional file 7: Table S6). *S-adenosylmethionine synthase* (*SAM*) plays an essential role in plant defense via catalyzing the production of S-adenosyl-L-methionine, which supplies as a methyl-group donor in the biosynthesis of numerous secondary metabolites. *SAMs* were observed to be down-regulated in leaves at 1 dpt, but were restored at 3dpt. Whereas, at 1 dpt, *SAMs* were mainly up-regulated by BTH in roots (Fig. 6, Additional file 7: Table S6). These results indicated that a wide range of enzymes participated in secondary metabolism pathways were induced by BTH, and their products may be contributed to BTH-dependent pathogen resistance.

### ROS metabolism-related genes that function in BTH response

In terms of ROS scavenging genes, a significant increase of peroxidases and polyphenol oxidases was observed at all time points in both leaves and roots (Fig. 6, Additional file 7: Table S6). Moreover, at 1 dpt, BTH induced up-regulation of a larger number of peroxidases in roots than in leaves. Among these DEGs, three peroxidases Ma6G21980, Ma9G30170 and Ma5G15750 were strongly up-regulated at 1 dpt in roots (Fold change > 100). Additionally, DEGs responsible for cell detoxification, such as *THIOREDOXIN* (*TRX*), *GLUTAREDOXIN* (*GRX*), *L-ascorbate oxidase* (*ASOD*), *L-ascorbate peroxidase* (*APX*) and *glutathione s-transferase* (*GST*) genes were differently modulated by BTH in the two tissues. Approximately 21.2% of up-regulated DEGs in roots exhibited a different expression pattern in leaves, where they were mainly suppressed by BTH. For example, the *TRX* genes Ma10G21720 and Ma3G25160 were up-regulated at 1 dpt and 3 dpt in roots (Fold change = 5.7 to 145.2), but were down-regulated in leaves. In roots, BTH primarily induced the majority of these DEGs at 1 dpt. However, a different trend was observed in leaves, as the numbers of up-regulated and down-regulated DEGs were similar (Fig. 6). The increases in the abundances of peroxidases, TRXs, GRXs and GSTs indicated a possible up-regulation of ROS scavenging capacity in banana plants under BTH treatment.

### Cell wall organization in BTH treatment

The plant cell wall acts as an important barrier against pathogen penetration into the intracellular space in plants [62]. Plant primary cell walls are mainly composed of polysaccharide such as pectins, celluloses and hemicelluloses, whereas secondary walls contain additional compounds such as lignins, cutin or wax [63]. Extensive research has revealed that activation of cell wall strengthening-related gene and induction of fungal cell wall degrading enzymes helps to inhibit pathogen entry [62]. In this study, BTH-treatment of banana plants induces the accumulation of a wide variety of proteins that are involved in cell wall metabolism at different levels in both leaves and roots, from remodeling and reinforcing of the plant cell wall to degradation of the pathogen itself. Among 254 DEGs responsible for cell wall organization, the *endoglucanase*, *chitinase* and *glucan endo-1,3-beta-glucosidase* associated with cell wall hydrolysis, and the *xyloglucan endotransglucosylase* (*XTH*), *polygalacturonase*, *pectinesterase*, *cellulose synthase*, *galacturonosyl transferase* and *expansin* involved in cell wall modification or maintenance were significantly modulated by BTH (Fig. 6, Additional file 7: Table S6). In particular, the abundance of most of these enzymes such as *polygalacturonase* and *glucan endo-1, 3-beta-glucosidase*, was found to be mainly accumulated in roots at 1 dpt (Fold change = 2.6 to 200.1), and only a few of these genes were affected at 3 dpt. In contrast, the numbers of up-regulated genes at 1 dpt and 3 dpt in leaves were similar (Fig. 6, Additional file 7: Table S6). The overall view for the cell wall metabolism genes showed a greater number of up-regulated than down-regulated genes at all time points. The result obtained implies that cell wall strengthening through structurally and chemically, especially in roots, may contribute to BTH-induced pathogen resistance in banana.

### Verification of the gene expression profiles by qRT-PCR

**Fig. 7:**
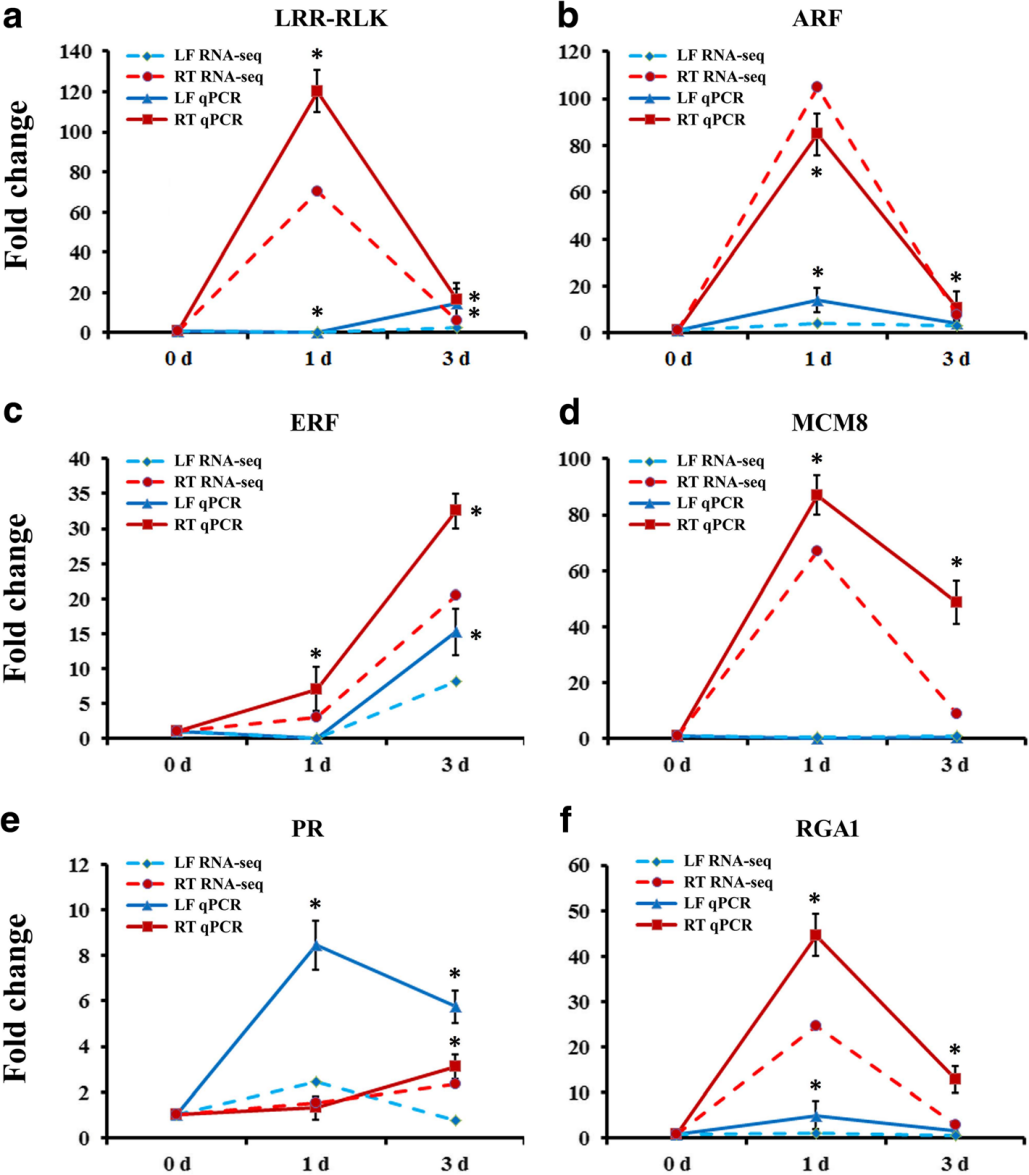
The expression profiles of 6 genes in banana leaves and roots by the quantitative RT-PCR. **a-f** The expression patterns of DEGs under BTH treatment by qPCR and RNA-Seq, respectively. *MaACTIN* was used as the reference gene. Data are presented as means ± SD of three independent assays. The asterisks indicate significant differences compared with the control treatment, *P < 0.01 by Student’s t-test. LF, leaf; RT, root

To verify the expression profiles of the genes in our analyses, we selected 6 DEGs for qRT-PCR using samples of leaves and roots originally used for RNA-Seq, which included the genes encoding *MaLRR-RLK* (Ma5G15040), *MaARF* (Ma4G22520), *MaERF* (Ma10G04450), *MaMCM8* (Ma11G08430), PR proteins (Ma9G16540) and *RGA1* (Ma6G19890). As shown in Fig. 7, all 6 genes showed the similar expression pattern as the in banana differential analysis results from RNA-Seq data. These DEGs were selected for their potentially key roles in regulating BTH signal transcription and pathogen response. For example, responses to biotic stimuli in *Arabidopsis* have often been reported to be associated with increased accumulation of transcripts encoding LRR-RLK proteins. *Brassinos teroid insensitive 1-associated receptor kinase 1* (*BAK1*) constituted one of the best studied plant LRR-RLK, and was shown to function as pattern recognition receptors mediating the recognition of microbial surface structures [64]. Additionally, the RGA proteins encoded by the majority of *R* genes represent important intracellular receptors that play specific roles in pathogens’ resistance [65]. Overexpression of pathogen-induced grapevine *VaRGA1* gene enhances disease resistance and abiotic stresses tolerance in *Nicotiana benthamian* [66]. The results showed that four genes were remarkably highly expressed in roots at 1 dpt, including *MaLRR-RLK*, *MaARF*, *MaMCM8* and *MaRGA1* (Fig. 7a, b, d and f), indicating their signal perception and transcription reactions after receiving the BTH signal. *MaERF* gene was more highly expressed at 3 dpt in the both leaves and roots (Fig. 7c), demonstrating that this gene may react slowly after BTH treatment. On the contrary, the expression level of the *PR* gene was higher in leaves at 1 and 3 dpt than in roots (Fig. 7e). The PR genes encode a class of proteins that suppress pathogens, detoxify toxins or virulence factors produced by pathogens and prevent pathogen advancement by enforcing plant cell walls. In *Nicotiana tabacum*, overexpression of *NtPR-Q* gene up-regulates multiple defense-related genes and enhances plant resistance to *Ralstonia solanacearum* [67]. Taken together, the results suggested that these genes might be involved in BTH-induced defense reactions of banana to Foc 4. Thus, the functions of these genes in banana should be further investigated in the future studies.

## Discussion

Two groups of BTH have been recognized as plant protection agents for a number of pathogens by inducing the plant’s own defense mechanisms in many plant species [18, 33–35]. Previously studies revealed that the plant resistance-inducing ability of BTH is based on mimicking the biological activation of SAR [34, 35]. In the present study, for the first time, we observed that the BTH-sprayed banana plants behave as more resistant to Foc 4 infection than unsprayed plants, without any direct toxic effect on plants growth and development. The primary goal of this study was to elucidate the defense mechanisms induced by BTH through investigating whole transcriptional changes of banana leaves and roots under control and BTH treatment. The RNA-Seq analysis carried out at 1 and 3 dpt of leaves and roots revealed that 6689 and 3624 genes were differentially modulated by BTH, respectively, as compared to the control. However, at all-time points, less than 12.4% of the DEGs were affected in both leaves and roots, and within each tested tissue, less than 9.8% of the DEGs were modulated at all of the times analyzed. This result indicated the excessive variability of such gene modulation over time and among tissues. After further functional classification of these DEGs, we observed that the defense responses were more dramatic in the roots than in the leaves. For example, enrichment analyses revealed the involvement of DEGs associated with the auxin signaling responses, cell cycle regulation, cell wall metabolism and defense signaling, were induced more intensely in the roots. This difference in genes expression was further supported by the change of the TFs activity and the change of RLKs content in the two tissues after BTH treatment. The results presented here have highlighted key processes that might further studied to improve BTH-induced resistance against Foc 4 infection, and also provided us with new tools with which to improve the disease resistance of banana and other plants.

Plant hormones such as SA, JA, auxin and ET, are well-known to play critical roles in plant-fungal interactions [14]. In general, SA and JA accumulation and signaling positively regulate defense to plant pathogenic fungi in a wide range of tested plants [16, 20, 68, 69]. ET signaling can either reduce or enhance disease symptoms caused by pathogen infection in an age-and/or condition-dependent manner among different species [14, 69]. In contrast, *F. oxysporum*-inoculated auxin-signaling or transport *Arabidopsis* mutants displayed prominently reduced symptoms compared to control plants, suggesting that local alterations of auxin levels are essential for *F. oxysporum* colonization [28]. Transcriptomic data obtained in this study revealed that the core auxin-signaling component such as *YUCCAs*, auxin efflux carriers (*PINs*) and *ARFs* were induced in root especially at the relatively early time points analyzed. Additionally, the expression levels of ACC synthases (*ACSs*), ethylene receptors (*ERSs*), EIN3-binding F-box protein (*EBFs*) and *ERFs* were significantly increased in leaves. An interesting feature about the activation by BTH was the differential tissue-specific response. Briefly, DEGs associated with ET signaling pathway were primarily modulated by BTH in leaves, whereas DEGs participate in auxin biosynthesis, transport and response were mainly induced in a root-specific manner. Previously studies on transcriptomic analysis of Foc 4 infected banana roots have revealed the differential expression of JA- and ET-related genes, but SA and auxin marker genes were unaffected [11, 37], indicating that in banana defense against Foc 4 is predominantly mediated via ET/JA signaling. In our study, transcriptome profiling of banana leaves and roots failed to detect activation of typical SA and JA marker genes in response to BTH, which is partially consistent with the previous researches [11, 32, 38]. These results led to the suggestion that BTH-induced Foc 4 resistance in banana was dependent on auxin and ET signals. However, how exactly BTH manipulates auxin and ET signaling in a tissue-specific manner remains to be addressed in the future.

In response to pathogen infection, plants develop systemic acquired resistance (SAR), a state of preparedness that provides elevated resistance during subsequent infections [70]. Besides pathogens, SA and its chemical analogs such as BTH are capable of inducing SAR in plants [34, 35]. Previously studies identified a number of compounds such as SA, JA, methyl salicylate, dihydroabetinal, azelaic acid, glycerol-3-phosphate, PR proteins and lipid transfer protein DIR1 as potential mobile signals of SAR [71, 72]. In BTH-sprayed banana roots, several *thaumatin-like proteins* (*TLPs*) genes and *lipid transfer proteins* (*LTPs*) were intensely induced at early stage of treatment. TLPs affect the fungus growth with β-glucanase and xylanase inhibitor activities, and have been reported against a variety of filamentous fungal pathogen [73]. Overexpression of *TLP* genes in transgenic plants have shown enhanced resistance and protection against different fungal pathogens [74]. Lipid transfer protein genes have also been shown to enhance plants resistance to trichothecene mycotoxin, in *Arabidopsis thaliana* and in *Saccharomyces cerevisiae* [75]. Additionally, genes encoding for nucleotide binding (NB)-leucine-rich repeat (LRR) disease resistance proteins (R proteins) such as RGA, RPM and RPP were found to be accumulated in both leaves and roots. R proteins have conserved domains and motifs that play specific roles in recognizing pathogens and activating inducible defenses. As these genes are associated to trigger disease resistance in various species, their enrichment amongst up-regulated genes might reflect traces of an enhanced immune response to Foc 4 infection of the BTH-treated plants.

The plant cell wall provides a structural framework to support plant growth and acts as a pathological and environmental barrier that defends against pathogens. Typical components of the cell wall include pectins, cellulose, hemicelluloses, lignins, proteins and water [63]. In response to an attack, plants deposit certain reinforcing polymers which may cause plant cell wall modification, and release degradation fragments to disturb fungal cell wall integrity [62]. Notably, in the present study, a great number of genes involved in cell wall metabolism including *endoglucanase*, *chitinase*, g*lucan endo-1,3-beta-glucosidase*, *polygalacturonase*, *pectinesterase*, *cellulose synthase*, *XTHs*, *galacturonosyl transferase* and *expansins* were found to increase in BTH-treated banana plants. It should be mentioned that chitinases and glucanases represent two large apoplastic protein families which are known to limit fungal growth via the degradation of the glucans from fungal cell walls [62]. Previous study has shown specific changes in the cell wall modification-related genes through transcriptomic and proteomic methods after the application of a specific biotic stress, and hypothesized that cell wall components may influence the interaction of pathogen and host. For instance, *Verticillium longisporum* infection of *Arabidopsis* leads to a specific increase of several extracellular proteins which mainly function in defense and cell wall metabolism [76]. The agroinfiltration of tobacco plants also results in the accumulation of a number of cell wall-modifying enzymes [77]. These results are consistent with similar findings for BTH-treated banana plants and might be common in plant pathogenesis. Early study also revealed that modified cell wall composition can indeed result in altered disease resistance phenotypes in host banana [78]. Therefore, the increase in the abundance of cell wall organization enzymes might explain the significant enhanced Foc 4 resistance after BTH treatment in banana.

RLKs play essential roles in plant immunity and development [49]. Plants employ a large number of RLKs as PRRs that detect PAMPs as the first layer of inducible defense [79]. In this study, a total of 107 and 124 RLK genes were induced in the leaves and roots of BTH-sprayed banana plants, respectively. Among these genes, 37 RLKs were mainly up-regulated in leaves, including 6 WAKL-, 4 cysteine-rich RLK-, 23 L/G-type lectin receptor kinase- and 4 lysM domain RLK-related genes. However, the other four types RLKs, which contain 6 serine/threonine receptor kinases, 25 receptor-like protein kinases, 78 LRR-RLKs and 6 proline-rich RLKs, were primarily elevated in roots. Previously, *ZmWAK*, which encodes a maize wall-associated kinase gene and confers quantitative resistance to head smut, was isolated by map-based cloning [80]. Lectin-RLKs are uniquely essential for plant disease resistance [49]. The *Arabidopsis* lectin S-domain-1 receptor-like kinase (LORE) acts as a receptor in sensing the lipid A moiety of bacterial LPSs (lipopolysaccharides), and contributes to plant immunity in response to bacteria [81]. LysM domain-RLKs represent a key class of receptors for microbial N-acetylglucosamine-containing glycans, which generally trigger defenses. A large portion of *Arabidopsis* LysM-RLKs have been shown to be essential for chitin-induced defenses in plants [49]. LRR-RLKs comprise the largest subgroup within the RLK superfamily in plants. Functional analysis of LRR-RLKs indicates that the majority of them function as regulators of defense response to viral, bacterial pathogen and necrotrophic fungus infection [49]. Therefore, the BTH-induced RLKs (particularly in roots) might play important roles in recognizing PAMPs produced by Foc 4 and in triggering defense responses.

**Fig. 8:**
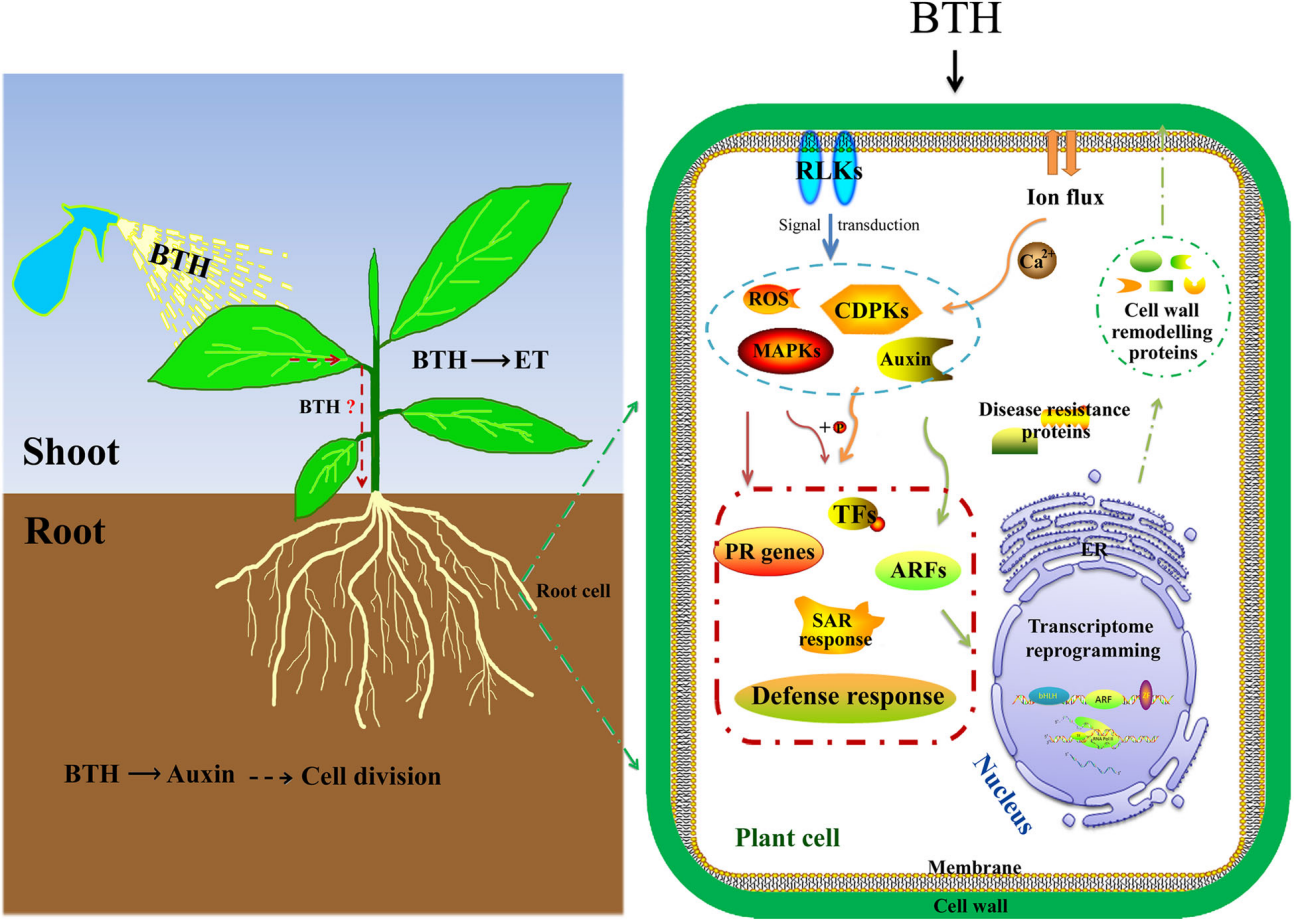
Putative model depicting BTH-mediated Foc 4 resistance via Ca^2+^, hormones and ROS signaling, according to the major DEGs that were modulated by BTH. ET, ethylene; RLKs, receptor-like kinases; ROS, reactive oxygen species; CDPKs, calcium-dependent protein kinases; MAPKs, mitogen-activated protein kinases; TFs, transcription factors; ARFs, auxin response factors; PR, pathogenesis related; SAR, systemic acquired resistance

Taken together, comparative analyses of transcriptomic data suggest that a different initial impact of BTH on shoots and roots (Fig. 8). More specifically, when BTH is applied to banana leaves, it primarily induces ET biosynthesis and response genes in leaves. Then, the BTH is somehow transported to the roots where the auxin signaling-related genes are uniquely up-regulated. The auxin signaling may subsequently affect cell division in roots, as underlined by the results of the enrichment analysis. We have highlighted in Fig. 8 the predominant influence of BTH on banana roots, considering that the soil-borne fungus Foc 4 mainly infects banana roots. The BTH-induced signals are perceived by membrane-located receptors, followed by hormones and Ca^2+^ transduction to activate downstream responsive genes, including TFs, PR genes, disease resistance and SAR-related genes. Furthermore, BTH altered the concentration of Ca^2+^ and ROS in the cytoplasm and elevated a series of ROS homeostasis related genes. The significantly altered TFs, such as *bHLHs*, *ARFs* and *ZFs* activate the expression of cell wall remodeling proteins, which may further affect cell wall organization and form a physical barrier that results in decreased Foc 4 infection.

## Conclusion

The current study delineates BTH-mediated endogenous biotic defense mechanisms adapted by banana plants under Foc 4 infection. A large number of DEGs associated with signal transduction, transcription activation/repression, disease resistant and ROS/hormone signaling pathways were identified in leaves and roots of BTH-sprayed plants. More importantly, the unique positive impacts of BTH on the cell cycle process and cell wall organization in roots haves also been observed. This ultimately may help to modify the structure of plant cell wall, which attributes a key role to protect plant roots from Foc 4 infection. Taking such insights into account, The DEGs identified in the present study could provide a resource for further developing resistant varieties against Foc 4 infection.

## Additional files

Additional file 1: **Table S7.** Gene-specific primers used for qRT-PCR. (XLSX 10 kb)

Additional file 2: **Table S1.** The information of 3996 identified banana novel protein-coding transcripts in the study. (XLSX 4797 kb)

Additional file 3: **Table S2a.** Expression information of DEGs in leaves at 1 day after BTH treatment. **Table S2b.** Expression information of DEGs in leaves at 3 day after BTH treatment. **Table S2c.** Expression information of DEGs in roots at 1 day after BTH treatment. **Table S2d.** Expression information of DEGs in roots at 3 day after BTH treatment. (XLSX 2340 kb)

Additional file 4: **Table S3.** GO terms of biological process enriched in banana leaves and roots. (XLSX 29 kb)

Additional file 5: **Table S4.** The significantly enriched pathways of DEGs in banana leaves and roots. (XLSX 11 kb)

Additional file 6: **Table S5.** Differentially expressed genes involved in protein kinases and transcription factors. (XLSX 225 kb)

Additional file 7: **Table S6.** Expression information of DEGs in Figs. 5 and 6. (XLSX 123 kb)

## Abbreviations

ABA: Abscisic acid
ANOVA: One-way analysis of variance
AP2/ERF: APETALA 2/Ethylene-Responsive Factor
APX: Ascorbate peroxidase
ARF: Auxin response factor
ASOD: Ascorbate oxidase
BABA: β -aminobutyric acid
bHLH: Basic helix-loop-helix
BTH: Benzothiadiazole
Ca2+: Calcium
CCOMT: Caffeoyl-CoA O-methyltransferase
Cdc20: Cell division cycle 20
cDNA: Complementary DNA
CDPKs: Calcium dependent protein kinases
COI1: Coronatine-Insensitive 1
COMT: Caffeic acid 3-O-methyltransferase
CSE: Caffeoylshikimate esterase
DEGs: Differentially expressed genes
DIR1: Defective in induced resistance 1
EBF: EIN3-binding F-box proteins
EIN2: Ethylene-insensitive 2
ERFs: Ethylene response factors
ET: Ethylene
ETI: Effector-triggered immunity
ETR1: Ethylene receptor 1
FDR: False discovery rate
FLS: Flavonol synthase caffeic acid
Foc 4: Fusarium oxysporum f. sp. cubense tropical race 4
FPKM: Fragments per kb exon model per million mapped fragments
GO: Gene ontology
GRX: Glutaredoxin
GST: Glutathione s-transferase
HR: Hypersensitive response
IFR: Isoflavone reductase
JA: Jasmonic acid
KEGG: Kyoto encyclopedia of genes and genomes
LDOX: Leucoanthocyanidin dioxygenase
LORE: Lectin S-domain-1 receptor-like kinase
LPSs: Lipopolysaccharides
LRR: Leucine-rich repeat
LTPs: Lipid transfer proteins
MADs: Mitotic spindle checkpoint proteins
MAPKs: Mitogen-activated protein kinases
NCBI: National center for biotechnology information
NPR1: Non-expression of pathogenesis related gene 1
Orc: Origin recognition complex subunits
PAMP: Pathogen-associated molecular pattern
PCNA: Proliferating cell nuclear antigen
PR: Pathogenesis-related
PRIs: Plant resistance inducers
PRR: Pattern recognition receptor
PTI: PAMP-triggered immunity
qRT-PCR: Quantitative real-time polymerase chain reaction
R protein: Disease-resistance protein
RLKs: Receptor-like kinases
RNA-Seq: Ribonucleic acid sequencing
ROS: Reactive oxygen species
SA: Salicylic acid
SAM: S-adenosylmethionine synthase
SAR: Systemic acquired resistance
SPL: Squamosa promoter binding-like protein
TFs: Transcription factors
TLPs: Thaumatin-like proteins
TRX: Thioredoxin
WAK: Wall-associated kinase
XTH: Xyloglucan endotransglucosylase
ZF: Zinc finger
